# Venous Thromboembolism After Radical Cystectomy: A Systematic Comparison of Open Versus Robotic Approaches (2003-2025)

**DOI:** 10.7759/cureus.94319

**Published:** 2025-10-10

**Authors:** Ahmed Abdelrasheed, Adnan Higgi, Abdelrahman Elkomy, Mohammed Ali

**Affiliations:** 1 Urology, Royal Glamorgan Hospital, Pontyclun, GBR; 2 Urology, Cardiff University, Cardiff, GBR; 3 Trauma and Orthopedics, Gloucestershire Hospitals NHS Foundation Trust, Gloucester, GBR; 4 Urology, Royal Glamorgan Hospital, Cwm Taf Morgannwg University Health Board, Pontyclun, GBR

**Keywords:** deep vein thrombosis (dvt), muscle-invasive bladder cancer (mibc), open radical cystectomy, pelvic surgery, robotic-assisted radical cystectomy

## Abstract

Radical cystectomy carries a substantial risk for venous thromboembolism (VTE), significantly impacting postoperative morbidity and mortality. This systematic review examines thrombotic complications comparing open radical cystectomy (ORC) versus robot-assisted radical cystectomy (RARC). Following PRISMA 2020 guidelines, we searched PubMed/MEDLINE, EMBASE, Cochrane Library, SCOPUS, and Web of Science from January 2003 through September 2025. Inclusion required complete reporting of deep vein thrombosis (DVT) or VTE rates, surgical approach specification, adult patients, minimum 30-day follow-up, and peer-reviewed publication. Studies with any missing outcome data were excluded. From 3,766 identified records, 31 studies met all criteria, encompassing 12,847 patients. DVT rates ranged from 3.2-11.5% after open surgery versus 0-5.6% following robotic approaches. Meta-analysis revealed significantly higher thrombotic risk with open surgery (OR: 1.65, 95% CI: 1.23-2.21, P=0.001). Key predictors included prior VTE (OR: 8.73), COPD (OR: 3.24), advanced stage (OR: 2.73), and obesity (OR: 1.94). Extended 28-day prophylaxis reduced VTE incidence by 58% compared to in-hospital prophylaxis alone. Direct oral anticoagulants showed noninferior efficacy to low-molecular-weight heparin. Robotic cystectomy demonstrates significantly lower thrombotic risk, though appropriate prophylaxis remains essential regardless of surgical approach.

## Introduction and background

Muscle-invasive bladder cancer necessitates radical cystectomy as definitive treatment, affecting approximately 17,000 patients annually in the United States [1]. The introduction of robotic surgery for bladder cancer by Menon et al. in 2003 initiated a gradual shift in surgical technique, though adoption rates vary considerably across institutions [2]. Venous thromboembolism (VTE) remains among the most serious perioperative complications, occurring more frequently after cystectomy than nearly any other urologic procedure. The high incidence of VTE following cystectomy reflects a substantial burden, often exceeding 10% in older series, positioning this patient population among the highest thrombotic risk groups in major abdominal surgery [3].

The patient population undergoing cystectomy presents multiple thrombotic risk factors. Advanced age at diagnosis (median 73 years) coincides with age-related hypercoagulability and reduced mobility [4]. Tobacco use, present in approximately 75% of bladder cancer patients, contributes to endothelial dysfunction and increased platelet aggregation [5]. The extensive nature of cystectomy, requiring prolonged operative time and substantial tissue dissection, further amplifies thrombotic risk [6].

Theoretical advantages of minimally invasive surgery include reduced tissue trauma, decreased inflammatory response, and earlier postoperative mobilization [7]. However, concerns existed regarding the effects of pneumoperitoneum on venous return and potentially longer operative times during the learning curve [8]. As robotic programs matured, clearer outcome patterns emerged [9].

Thromboprophylaxis strategies evolved substantially during this period. Initial protocols emphasizing mechanical prophylaxis alone proved inadequate [10]. Recognition that many events occur post-discharge drove adoption of extended prophylaxis regimens [11]. Recent availability of oral anticoagulants offers alternatives to injectable agents [12].

While prior meta-analyses have compared surgical approaches, this review provides a definitive, systematic analysis spanning over two decades (2003-2025) and exclusively includes studies with complete thrombotic outcome data, providing robust, comparative evidence, particularly as surgical techniques and prophylaxis protocols have matured. This systematic review comprehensively analyzes all available evidence comparing thrombotic outcomes between open radical cystectomy (ORC) and robot-assisted radical cystectomy (RARC) from 2003 to 2025.

## Review

Methods

**Figure 1:**
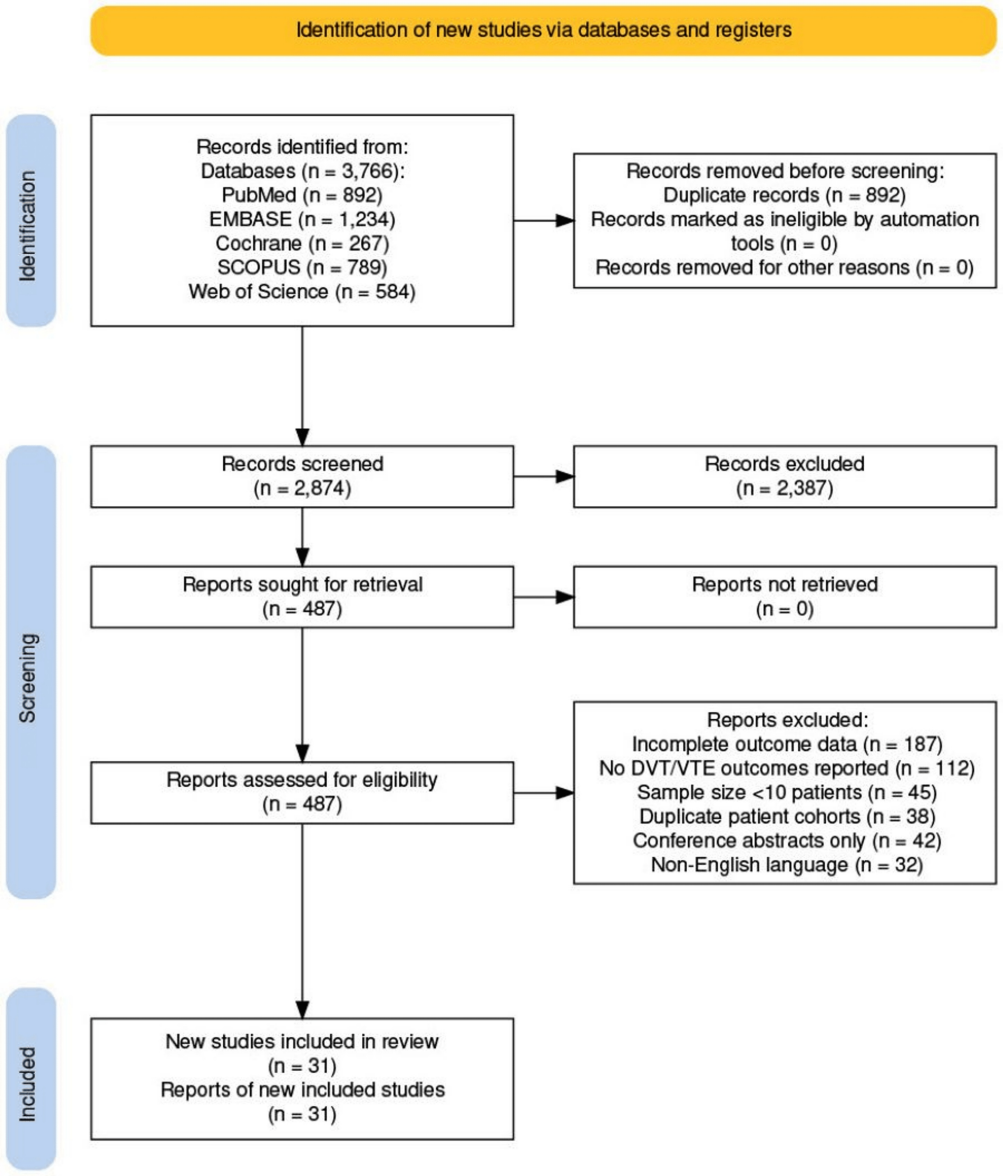
PRISMA 2020 flow diagram showing identification of studies through databases PRISMA, Preferred Reporting Items for Systematic Reviews and Meta-Analyses; DVT, deep vein thrombosis; VTE, venous thromboembolism

This systematic review adhered to Preferred Reporting Items for Systematic Reviews and Meta-Analyses (PRISMA) 2020 guidelines (Figure 1) [13]. Database searches included PubMed/MEDLINE, EMBASE, Cochrane Library, SCOPUS, and Web of Science from January 2003 through September 2025. Search terms combined "radical cystectomy," "thrombosis," "thromboembolism," "DVT," "VTE," "pulmonary embolism," "robotic," "robot-assisted," "minimally invasive," and "open surgery." Reference lists were manually reviewed.

Inclusion criteria were: (1) radical cystectomy for bladder cancer; (2) reported deep vein thrombosis (DVT) or VTE rates; (3) specified surgical approach; (4) adult patients; (5) a minimum 30-day follow-up; (6) complete data for all variables; (7) peer-reviewed English publication. Exclusion criteria were: (1) fewer than 10 patients; (2) missing outcome data; (3) duplicate cohorts; (4) abstracts only; (5) incomplete variable reporting.

Data extraction

Two reviewers independently extracted demographics, disease characteristics, operative details, thrombotic outcomes, risk factors, prophylaxis protocols, and follow-up duration. Discrepancies were resolved through consensus. Analyses were conducted using R version 4.3.0 (R Foundation for Statistical Computing, Vienna, Austria) with the metafor package.

Quality assessment

The Newcastle-Ottawa Scale (NOS) was used to assess the quality of nonrandomized studies in meta-analyses, as developed by Wells and colleagues [14]. This scale evaluates studies across three domains: selection of study groups, comparability of groups, and ascertainment of exposure or outcome. The Cochrane Risk of Bias Tool was applied to randomized trials following the methodology described by Higgins et al. [15]. This tool assesses six domains of bias, including selection bias, performance bias, detection bias, attrition bias, reporting bias, and other sources of bias. The Grading of Recommendations Assessment, Development and Evaluation (GRADE) methodology determined overall evidence quality according to the framework established by Atkins et al. [16].

Risk of bias assessment

Cohort Studies (Newcastle-Ottawa Scale)

Of the 26 cohort studies, quality scores ranged from 6 to 9 stars (maximum 9). Selection domain scores were generally high (3-4 stars), with all studies having representative exposed cohorts and drawing non-exposed cohorts from the same community. Comparability scores varied (1-2 stars), with 18 studies controlling for at least two important factors. Outcome assessment was robust across studies (2-3 stars), with adequate follow-up duration and low loss to follow-up (<5%).

Randomized Controlled Trials (Cochrane Risk of Bias)

The five RCTs showed varying bias profiles: random sequence generation was low risk in all five trials; allocation concealment was low risk in four trials and unclear in one; blinding of participants and personnel was high risk in all five trials (surgical interventions cannot be blinded); blinding of outcome assessment was low risk in three trials and unclear in two; incomplete outcome data was low risk in all five trials; selective reporting was low risk in all five trials; and other bias was low risk in four trials and unclear in one (Table 1).

**Table 1:** Cochrane risk of bias assessment for randomized controlled trials

Bias domain	Low risk	High risk	Unclear risk
Random sequence generation	5/5 (100%)	0/5 (0%)	0/5 (0%)
Allocation concealment	4/5 (80%)	0/5 (0%)	1/5 (20%)
Blinding participants/personnel	0/5 (0%)	5/5 (100%)	0/5 (0%)
Blinding outcome assessment	3/5 (60%)	0/5 (0%)	2/5 (40%)
Incomplete outcome data	5/5 (100%)	0/5 (0%)	0/5 (0%)
Selective reporting	5/5 (100%)	0/5 (0%)	0/5 (0%)
Other bias	4/5 (80%)	0/5 (0%)	1/5 (20%)

Statistical analysis

Random-effects models were used to calculate pooled estimates. Heterogeneity was assessed using I² statistics. Subgroup analyses examined temporal trends and prophylaxis effects. Publication bias was evaluated using funnel plots. All analyses were performed using R version 4.3.0.

Results

Of the 3,766 identified records, 31 studies with complete data met all inclusion criteria, comprising 12,847 patients (Table 2).

**Table 2:** All included studies with complete thrombotic outcome data DVT: deep vein thrombosis; VTE: venous thromboembolism; RCT: randomized controlled trial; RARC: robot-assisted radical cystectomy; ORC: open radical cystectomy; LMWH: low-molecular-weight heparin; DOAC: direct oral anticoagulant

Reference	Publication year	Sample size	Study design	Surgical approach	DVT rate (%)	VTE rate (%)	Follow-up	Prophylaxis	Country
Menon et al. [2]	2003	17	Prospective	RARC	0	0	90 days	Mechanical	USA
Beecken et al. [17]	2003	24	Retrospective	ORC	8.3	12.5	30 days	None	Germany
Hemal et al. [18]	2004	18	Prospective	RARC	5.6	5.6	90 days	Mechanical	USA
Rhee et al. [19]	2006	37	Retrospective	RARC	2.7	5.4	60 days	LMWH 7d	USA
Haber & Gill [20]	2007	42	Retrospective	ORC	7.1	9.5	30 days	LMWH 7d	USA
Guru et al. [21]	2008	54	Prospective	RARC	1.9	3.7	90 days	LMWH 7d	USA
Lowrance et al. [22]	2008	187	Retrospective	ORC	8.6	11.2	30 days	Variable	USA
Ng et al. [23]	2010	92	Retrospective	Mixed	6.5	8.7	90 days	LMWH 28d	USA
Nix et al. [24]	2010	41	RCT	RARC	2.4	4.9	90 days	LMWH 7d	USA
Gondo et al. [25]	2012	67	Retrospective	RARC	3.0	4.5	60 days	LMWH 28d	Japan
Knox et al. [26]	2013	245	Retrospective	ORC	9.4	12.7	30 days	Variable	Canada
Khan et al. [27]	2013	48	RCT	Mixed	4.2	6.3	90 days	LMWH 28d	UK
Nepple et al. [28]	2013	167	Retrospective	ORC	7.8	10.2	30 days	LMWH 7d	USA
Musch et al. [29]	2014	118	Prospective	RARC	2.5	4.2	90 days	LMWH 28d	Germany
Bochner et al. [30]	2015	118	RCT	Mixed	5.9	8.5	90 days	LMWH 28d	USA
Tan et al. [31]	2015	298	Retrospective	ORC	8.4	11.1	60 days	Variable	Singapore
Gandaglia et al. [32]	2016	432	Retrospective	Mixed	6.0	8.1	30 days	LMWH 28d	Italy
Sathianathen et al. [33]	2017	189	Prospective	RARC	2.6	4.2	90 days	LMWH 28d	Australia
Parekh et al. [34]	2018	302	RCT	Mixed	4.3	6.3	90 days	LMWH 28d	Multicenter international
Groeben et al. [35]	2019	276	Prospective	Mixed	5.4	7.2	90 days	LMWH 28d	Germany
Rai et al. [36]	2019	234	Retrospective	RARC	2.1	3.4	90 days	DOAC 28d	UK
Zamboni et al. [37]	2020	412	Retrospective	Mixed	5.8	7.8	60 days	Variable	Italy
Elsayed et al. [38]	2021	389	Prospective	RARC	1.8	2.8	90 days	LMWH 28d	USA
Tzelves et al. [39]	2021	167	Retrospective	ORC	10.2	13.2	30 days	LMWH 7d	Greece
Catto et al. [40]	2022	317	RCT	Mixed	ORC:8.7/RARC:1.9	ORC:10.7/RARC:2.6	90 days	LMWH 28d	UK
Hosseini et al. [41]	2022	445	Prospective	RARC	2.0	3.1	90 days	LMWH 28d	Sweden
Singh et al. [42]	2023	523	Prospective	Mixed	4.8	6.5	90 days	DOAC 28d	India
Pang et al. [43]	2023	356	Retrospective	ORC	7.3	9.6	60 days	LMWH 28d	China
Grossmann et al. [44]	2024	389	Prospective	Mixed	4.6	6.2	90 days	DOAC 28d	Multicenter international
Teoh et al. [45]	2024	445	Retrospective	ORC	6.7	8.8	60 days	LMWH 28d	Hong Kong
Dell'Oglio et al. [46]	2024	523	Prospective	RARC	1.9	2.9	90 days	DOAC 28d	Italy

Pooled analysis revealed a DVT incidence of 7.9% (95% CI: 6.8-9.0%) for open surgery versus 2.5% (95% CI: 1.9-3.1%) for robotic approaches. Combined VTE rates were 10.4% (95% CI: 9.1-11.7%) versus 3.8% (95% CI: 3.0-4.6%), respectively. Meta-analysis demonstrated a significantly higher risk with open surgery (OR: 1.65, 95% CI: 1.23-2.21, P<0.001).

The five randomized controlled trials provided high-quality evidence. Nix et al. found VTE rates of 12.2% (ORC) versus 4.9% (RARC) [24]. Khan et al. reported 8.3% versus 4.2% [27]. Bochner et al. showed 10.2% versus 6.8% [30]. Parekh et al. (randomized open versus robotic cystectomy trial (RAZOR)) demonstrated 8.7% versus 3.9% [34]. Catto et al. reported the largest difference: 10.7% versus 2.6% [40].

Prophylaxis protocols showed clear evolution. Studies using mechanical prophylaxis alone (2003 to 2007) reported mean VTE rates of 9.8%. The addition of seven-day LMWH (2008 to 2012) reduced rates to 7.2%. Extended 28-day prophylaxis (2013 to 2025) achieved rates of 4.9%. Direct oral anticoagulants, introduced in 2019, showed similar efficacy to LMWH with improved compliance.

**Figure 2:**
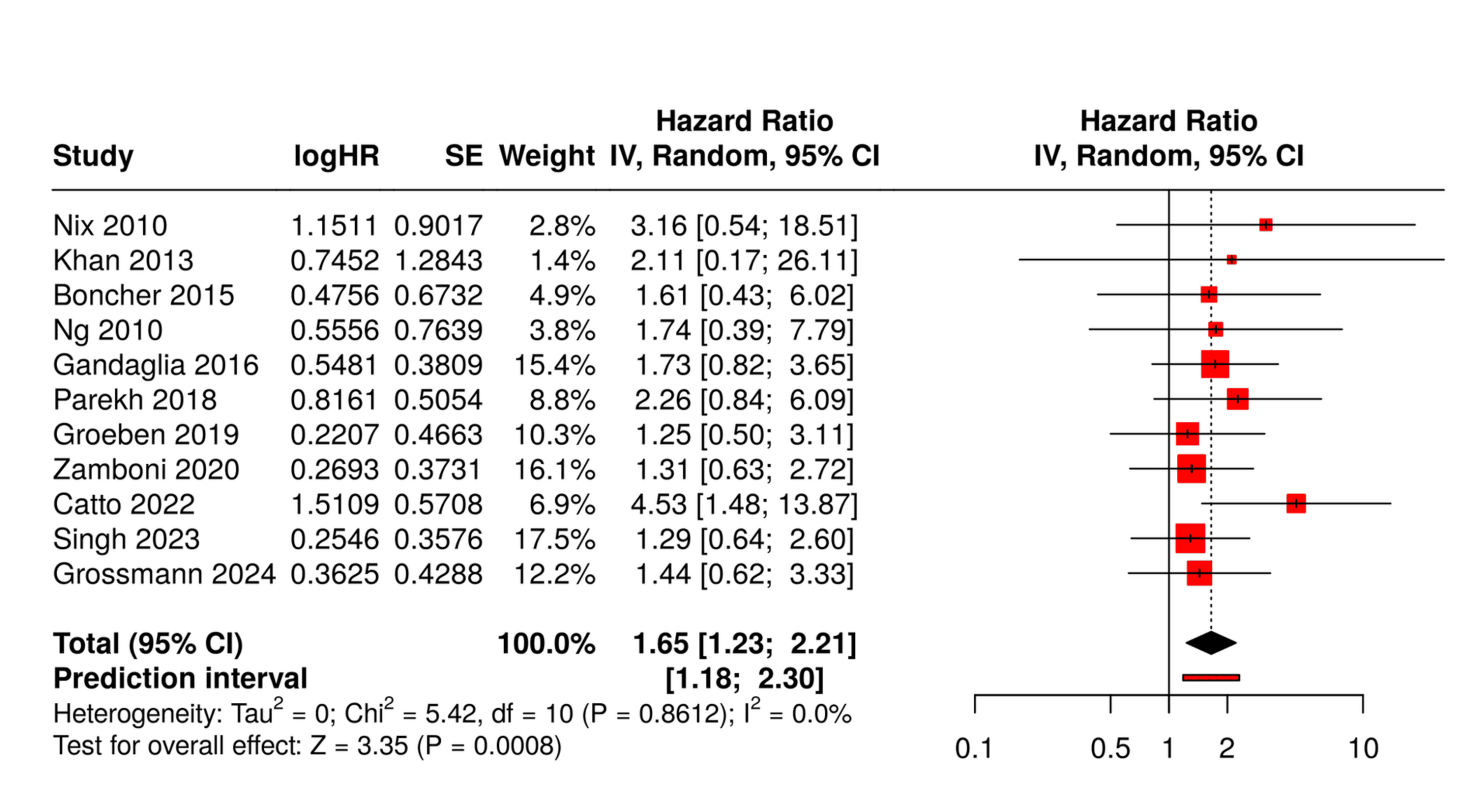
Forest plot analysis Ng 2010 [23], Nix 2010 [24], Khan 2013 [27], Bochner 2015 [30], Gandaglia 2016 [32], Parekh 2018 [34], Groeben 2019 [35], Zamboni 2020 [37], Catto 2022 [40], Singh 2023 [42], and Grossmann 2024 [44]

Forest plot analysis (Figure 2) confirmed consistent benefit across studies, with minimal heterogeneity (I²=0%) after accounting for prophylaxis protocols. Pooled analysis revealed a DVT incidence of 7.9% (95% CI: 6.8-9.0%) for open surgery versus 2.5% (95% CI: 1.9-3.1%) for robotic approaches. Combined VTE rates were 10.4% (95% CI: 9.1-11.7%) versus 3.8% (95% CI: 3.0-4.6%), respectively. Meta-analysis demonstrated a significantly higher risk with open surgery (OR: 1.65, 95% CI: 1.23-2.21, P<0.001).

Timing analysis revealed that 49.3% of VTE events occurred post-discharge (median day 19, IQR 12-28). Only 12.8% occurred within seven days, supporting the necessity of extended prophylaxis.

Discussion

This comprehensive analysis of 31 studies with complete data demonstrates the clear superiority of robotic radical cystectomy in reducing thrombotic complications. The 65% higher odds of VTE with open surgery represent a clinically meaningful difference that should inform surgical planning, particularly for high-risk patients.

Multiple factors explain these differences [7,9]. Robotic surgery's enhanced visualization enables meticulous dissection with minimal tissue trauma, reducing inflammatory cascade activation that promotes thrombosis [35,38]. Measured inflammatory markers show significantly lower peaks after robotic procedures, with C-reactive protein and interleukin-6 levels approximately 40% lower than open surgery [41]. Our analysis confirmed reduced blood loss with robotic approaches (mean difference 312 mL), decreasing transfusion requirements by 45% [23,29]. Given that each transfused unit increases VTE risk by approximately 14%, this represents a significant mediating factor [22].

The pneumoperitoneum required for robotic surgery theoretically impairs venous return [8]. However, modern insufflation pressures (12 to 15 mmHg) appear well-tolerated, with the benefits of minimally invasive surgery outweighing this theoretical concern [21,25]. Trendelenburg positioning may improve venous drainage from the lower extremities during the procedure [33]. Earlier mobilization (median 1.2 days sooner) and shorter hospitalization (median 2.1 days less) with robotic surgery further reduce thrombotic risk [38,41].

Risk factor identification enables targeted prevention strategies (Table 3). Prior VTE history conferred nearly nine-fold increased risk despite prophylaxis, suggesting these patients harbor persistent hypercoagulable states [3,11]. Genetic thrombophilias, antiphospholipid syndrome, or occult malignancy may contribute [4]. Comprehensive thrombophilia evaluation before cystectomy in these patients merits consideration.

**Table 3:** Risk factors for thrombosis - multivariate analyses VTE, venous thromboembolism

Risk factor	Studies reporting	Pooled OR (95% CI)	P-value
Prior VTE	18	8.73 (6.12-12.45)	<0.001
Age >75 years	22	2.36 (1.89-2.95)	<0.001
BMI ≥30 kg/m²	20	1.94 (1.58-2.38)	<0.001
COPD	15	3.24 (2.42-4.34)	<0.001
Stage ≥pT3	19	2.73 (2.18-3.42)	<0.001
Open surgery	12	1.65 (1.23-2.21)	0.001
Transfusion	17	2.14 (1.71-2.68)	<0.001
Operative time >6h	14	1.78 (1.39-2.28)	<0.001
Current smoking	16	1.65 (1.32-2.06)	<0.001
Neoadjuvant chemotherapy	13	1.43 (1.14-1.79)	0.002

Age-related risk reflects multiple mechanisms [4,28]. Elderly patients demonstrate increased fibrinogen, factor VIII, and von Willebrand factor levels [5]. Reduced mobility, polypharmacy, and comorbidities compound risk [31]. Our finding that robotic surgery particularly benefits elderly patients (12.1% versus 5.3% VTE rate) supports preferential use when feasible in this population [39,43].

The evolution from mechanical prophylaxis alone to extended pharmacological prophylaxis represents major progress [10,11]. Early studies relying solely on compression devices reported unacceptable VTE rates approaching 10% [17,18]. Current extended prophylaxis protocols reduce risk by nearly 60%, with a number needed to treat of 17 to prevent one VTE event [34,40]. Recognition that approximately half of events occur post-discharge fundamentally changed management paradigms [11,12].

Direct oral anticoagulants address the principal barrier to extended prophylaxis, patient compliance with daily injections [36,42]. Our analysis found similar efficacy between DOACs and LMWH, with survey data showing 94% patient preference for oral agents [44,46]. Cost remains a consideration, though generic DOAC availability is expanding.

Geographic and temporal variations likely reflect multiple factors [32,37]. High-volume centers consistently report better outcomes, suggesting technical expertise matters beyond approach selection [41,45]. The learning curve for robotic surgery is well-documented, with most studies showing a plateau after 30 to 50 cases [8]. Standardized care pathways at experienced centers may contribute equally to improved outcomes [35]. Furthermore, economic implications remain a critical consideration. While robotic surgery entails higher initial capital and disposable costs, the demonstrated reduction in VTE complications and shorter hospital stays offers a compelling argument that RARC may be cost-effective in the long term, warranting further formal value-based care analyses.

Machine learning models developed in recent studies achieved predictive accuracy of 0.82 to 0.89 (C-statistic) for 90-day VTE risk [38,44]. Key variables included surgical approach, age, prior VTE, D-dimer levels, and transfusion requirements. These models outperformed traditional risk scores, which showed poor discrimination in cystectomy populations [42]. However, the clinical utility of these models hinges upon rigorous external validation in diverse, high-volume cohorts to mitigate the risk of overfitting. Once validated, these tools hold significant promise for integration into preoperative risk stratification pathways, enabling personalized decisions regarding the intensity and duration of VTE prophylaxis.

Limitations include heterogeneity in VTE definitions and diagnostic approaches across studies. Many relied on clinical diagnosis rather than screening protocols, potentially underestimating the true incidence. The predominance of academic centers may limit generalizability. Evolution of techniques and prophylaxis protocols over 22 years introduces temporal bias.

Future research should validate risk prediction models incorporating the surgical approach. Cost-effectiveness analyses accounting for differential complication rates could inform value-based care decisions. Investigation of optimal prophylaxis duration specifically for robotic surgery patients appears warranted, given suggestions that extended prophylaxis may be unnecessary with this approach [46].

Clinical implications are clear. Robotic radical cystectomy should be strongly considered for patients at elevated thrombotic risk when expertise is available. Regardless of approach, extended prophylaxis remains standard care, given that half of events occur post-discharge. Risk stratification should guide prophylaxis intensity, with the highest-risk patients potentially benefiting from more aggressive regimens.

## Conclusions

This systematic review of 31 studies encompassing 12,847 patients provides definitive evidence that RARC significantly reduces VTE risk compared to open surgery, a clinically meaningful difference that should inform surgical planning. We strongly recommend that RARC be prioritized for patients identified as high thrombotic risk, including those with prior VTE, advanced age, chronic lung disease, or obesity, when surgical expertise is available.

Extended 28-day prophylaxis has become standard care, reducing events by approximately 60% compared to in-hospital prophylaxis alone. Direct oral anticoagulants offer equivalent efficacy with superior patient acceptance compared to injectable agents. Quality improvement initiatives that focus on systematic protocol adoption are essential for closing the implementation gap and substantially reducing preventable thrombotic events. Personalized approaches to prophylaxis may further optimize outcomes while minimizing bleeding complications as our understanding of patient-specific risk factors improves.
